# Near‐infrared spectroscopy detects age‐related differences in skeletal muscle oxidative function: promising implications for geroscience

**DOI:** 10.14814/phy2.13588

**Published:** 2018-02-07

**Authors:** Susie Chung, Ryan Rosenberry, Terence E. Ryan, Madison Munson, Thomas Dombrowsky, Suwon Park, Aida Nasirian, Mark J. Haykowsky, Michael D. Nelson

**Affiliations:** ^1^ Applied Physiology and Advanced Imaging Laboratory Department of Kinesiology University of Texas at Arlington Arlington Texas; ^2^ Department of Physiology East Carolina University Greenville North Carolina; ^3^ College of Nursing University of Texas at Arlington Arlington Texas

**Keywords:** Aging, near infrared spectroscopy, oxidative capacity

## Abstract

Age is the greatest risk factor for chronic disease and is associated with a marked decline in functional capacity and quality of life. A key factor contributing to loss of function in older adults is the decline in skeletal muscle function. While the exact mechanism(s) remains incompletely understood, age‐related mitochondrial dysfunction is thought to play a major role. To explore this question further, we studied 15 independently living seniors (age: 72 ± 5 years; m/f: 4/11; BMI: 27.6 ± 5.9) and 17 young volunteers (age: 25 ± 4 years; m/f: 8/9; BMI: 24.0 ± 3.3). Skeletal muscle oxidative function was measured in forearm muscle from the recovery kinetics of muscle oxygen consumption using near‐infrared spectroscopy (NIRS). Muscle oxygen consumption was calculated as the slope of change in hemoglobin saturation during a series of rapid, supra‐systolic arterial cuff occlusions following a brief bout of exercise. Aging was associated with a significant prolongation of the time constant of oxidative recovery following exercise (51.8 ± 5.4 sec vs. 37.1 ± 2.1 sec, *P* = 0.04, old vs. young, respectively). This finding suggests an overall reduction in mitochondrial function with age in nonlocomotor skeletal muscle. That these data were obtained using NIRS holds great promise in gerontology for quantitative assessment of skeletal muscle oxidative function at the bed side or clinic.

## Introduction

Age is the greatest risk factor for chronic disease, and is associated with a marked decline in functional capacity and quality of life (Niccoli and Partridge 2012; Torre et al. 2015; Jakovljevic 2017). A key factor contributing to loss of function in aging is the decline in skeletal muscle function (Buskirk and Hodgson 1987; McCarter 1990; Brooks and Faulkner 1994). While the exact mechanism(s) remains incompletely understood, age‐related mitochondrial dysfunction is thought to play a major role (Coggan et al. 1993; McCully et al. 1993; Papa 1996; Kerner et al. 2001; Chabi et al. 2008; Marzetti et al. 2008; Gouspillou et al. 2014; Hepple 2014).

Until recently, the assessment of mitochondrial function has been limited to invasive muscle biopsies and/or expensive and time consuming magnetic resonance spectroscopy (MRS) techniques, which limit patient access. Functional evaluation of skeletal muscle oxidative metabolism has also been assessed by pulmonary oxygen uptake (Grassi et al. 1996; Rossiter et al. 1999); however, this assessment is indirect, and technically challenging. Recent advancements in near‐infrared spectroscopy (NIRS), however, have provided a robust, clinical platform to noninvasively assess muscle oxygen consumption/oxidative function across a wide range of muscles and disease states (McCully et al. 2011; Brizendine et al. 2013; Erickson et al. 2013; Ryan et al. 2013a,b,c, 2014a,b; Southern et al. 2014, 2015; Adami and Rossiter 2017). For a detailed review, readers are directed to (Grassi and Quaresima 2016; Willingham and McCully 2017). This novel approach utilizes a series of rapid, supra‐systolic arterial cuff occlusions to measure postexercise muscle oxygen consumption recovery kinetics (i.e., mitochondrial function), which is analogous to the recovery of phosphocreatine using ^31^P‐MRS (Ryan et al. 2013c). Whether this approach can be used to assess age‐related mitochondrial dysfunction remains untested. Addressing this fundamental question is critical to advance current clinical assessment practices and/or the design of large clinical trials focused on age‐related diseases.

The purpose of this study was therefore to compare skeletal muscle oxidative function between two distinct age groups (<35 vs. >65 years) using the aforementioned NIRS approach. We hypothesized that aging would prolong postexercise muscle oxygen consumption recovery kinetics (i.e., mitochondrial function) compared to young individuals, demonstrating the clinical utility of this novel imaging approach.

## Methods

### Participants

A total of 39 participants between the ages of 20 and 80 years of age were recruited from the local Dallas‐Fort Worth community. The participants were divided into two groups based on age: young (18–30 years) and elderly (60–85 years). None of the young participants had any history of cardiovascular, metabolic, or neurological disease. Some of the aging individuals had history of hypertension (*n* = 12) and hypercholesterolemia (*n* = 10); however, none had overt heart, metabolic, or neurological disease, or were current smokers. All subjects presented to the lab in a fasted state, having abstained from alcohol, caffeine, and vigorous exercise for at least 24 h. All subjects provided written informed consent before being enrolled to participate in this study. The study was approved by the Institutional Review Board at the University of Texas at Arlington, and conformed to the standards set by the latest version of *the Declaration of Helsinki*.

Activity level was self‐reported. Subjects were asked if they performed regular exercise, as well as frequency, duration, and type. Activity level was then quantified by multiplying the frequency by duration and reported as minutes per week. The intensity of exercise was rated as follows: walking was defined as mild‐to‐moderate, whereas cycling or running was defined as moderate‐to‐vigorous.

### Experimental protocol

Postexercise muscle oxygen consumption (mVO_2_) was assessed as previously described (Ryan et al. 2013c, 2014a,b). Briefly, each subject was positioned in the supine position with their nondominant hand extended to comfortably reach a handgrip dynamometer (Stoelting 56380; Stoelting Co., Wheat Lane, IL). A noninvasive dual‐wavelength NIRS optode (OxiPlex TS, Model 95205; ISS, Champaign, IL) was placed longitudinally over the muscle belly of the flexor digitorum profundus, the main muscle responsible for handgrip exercise. This nonlocomotor muscle was chosen to avoid influence of lower limb activity discrepancies likely found in these two populations (i.e., young vs. elderly). Oxyhemoglobin and deoxyhemoglobin concentration was measured using a single channel consisting of eight laser diodes emitting at wavelengths of 690 and 830 nm (four at each wavelength). The laser diodes and photomultiplier were contained in a light plastic probe consisting of two parallel rows of emitter fibers and one detector fiber bundle comprising source‐detector separations of 2.0, 2.5, 3.0, and 3.5 cm for both wavelengths. The frequency modulation of laser intensity was 100 MHz. The NIRS optode was placed firmly against the skin, held in place with a Velcro strap, and then encased in a black cloth to block the entry of light near the optical sensor. A blood pressure cuff (Hokanson SC5, D. E. Hokanson Inc, Bellevue, WA) was placed on the upper arm of the exercising hand; the cuff was powered by a rapid cuff inflator (Hokanson E20).

**Figure 1:**
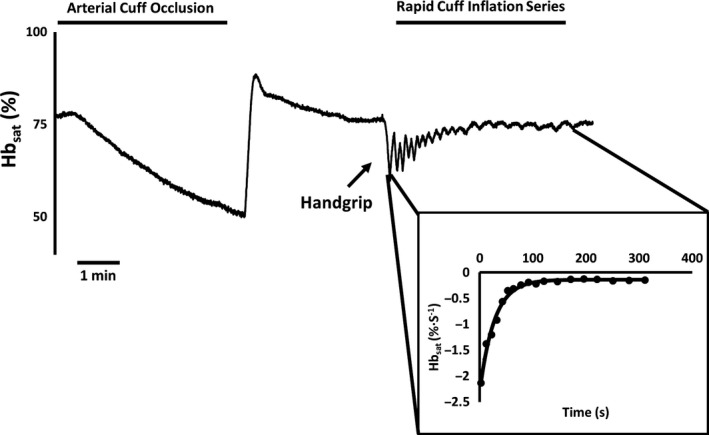
Representative data tracing from an individual subject showing a typical muscle oxygenation test. Oxygenated and deoxygenated hemoglobin were measured by near‐infrared spectroscopy over the flexor digitorum profundus. Data are reported as oxyhemoglobin saturation (i.e., Hb_sat_). Each test began with a brief baseline period, followed by a 5 min cuff occlusion in order to establish the desaturation reserve. Subjects then performed a brief bout of isometric handgrip exercise at 50% of maximal voluntary contraction, followed by a series of rapid cuff occlusions. The slope of each postexercise cuff occlusion was then measured, plotted against time, and fit to a monoexponential equation to calculate the muscle oxygen consumption recovery kinetics (as shown in the data insert).

As illustrated in Figure 1, following a brief baseline period, a 5 min arterial cuff occlusion was applied in order to establish each subject's desaturation reserve. After stabilization of oxygenated and deoxygenated hemoglobin, participants were instructed to perform a brief bout of isometric handgrip exercise (at 50% of each individual's maximal voluntary contraction, or MVC) until muscle oxygen saturation dropped by ~50% (~10–30 sec). After exercise cessation, the following series of rapid cuff inflations were employed in order to form an mVO_2_ recovery curve: 5 sec on/5 sec off for inflations #1–6, 7 sec on/7 sec off for inflations #7–10, 10 sec on/15 sec off for inflations #11–14, and 10 sec on/20 sec off for inflations #15–18. Once oxygenated and deoxygenated hemoglobin levels returned to baseline levels (typically between 2 and 5 min), the protocol was repeated and the average of at least two tests was reported.

### Calculation of mVO_2_ and its recovery rate

The calculation of mVO_2_ was expressed by the slope of change in the Hb_sat_ signal (oxygenated hemoglobin + myoglobin [O_2_Hb]/(oxygenated hemoglobin + myoglobin [O_2_Hb] + deoxygenated hemoglobin + myoglobin [HHb]) × 100). The postexercise recovery measurements of mVO_2_ were fit to the following monoexponential curve, as previously described (Ryan et al. 2013c, 2014a,b) using commercially available software (OriginPro, OriginLab, Corp., Northampton, MA):

$$y=\text{End}-\Delta \times e^{-kt},$$

where “*y*” is the relative mVO_2_ during cuff inflation, “End” represents the mVO_2_ value immediately following the cessation of exercise; delta (“*Δ*”) signifies the change in mVO_2_ from rest to the end of exercise; “*k*” is the fitting rate constant; “*t*” is time. The initial rate of oxygen consumption was defined as the first measurement immediately after exercise cessation.

### Statistical analyses

Statistical analysis was performed using SigmaPlot 13.0 (Systat Software, Inc. San Jose, CA). Data are expressed as a mean ± standard error unless otherwise specified. Normally distributed data were compared using a Student's *t*‐test. If data were not normally distributed, a Mann–Whitney Rank Sum Test was used to compare group differences. Statistical analysis was not performed on activity level given the gross differences in sample size for the three predetermined exercise intensities.

## Results

Of the 39 participants recruited, data analysis was possible in all but seven of the subjects. Data from five elderly participants and two young participants were excluded due to technical limitations (*n* = 3) or poor NIRS data quality (*n* = 4). Individual characteristics for the 32 who successfully completed the study are shown in Table 1. There were no adverse events or contraindications to testing.

**Table 1:** Subject characteristics

	Young	Old	*P*‐value
*n*	17	15	–
Male/female	8/9	4/11	–
Age (years)	25 ± 4	72 ± 5	<0.001
Height (cm)	167.4 ± 12.3	168.1 ± 9.9	0.85
Weight (kg)	69.5 ± 16.0	77.7 ± 15.3	0.126
BMI	24.5 ± 3.3	27.6 ± 6.0	0.05
MVC (kg)	35.1 ± 13.0	26.0 ± 8.4	<0.001
Activity level			
Sedentary, min/week (*n*)	– (1)	– (0)	–
Mild to moderate, min/week (*n*)	120 ± 79 (3)	182 ± 39 (11)	–
Moderate to vigorous, min/week (*n*)	225 ± 217 (13)	336 ± 289 (4)	–
Medications			
*β*‐blocker (*n*)	–	1	–
ACE‐inhibitor (*n*)	–	2	–
ARB (*n*)	–	3	–
Thyroid hormone (*n*)	–	3	–
Statin (*n*)	–	3	–
NSAIDs/blood thinners (*n*)	–	5	–
Immunosuppressant (*n*)	–	1	–
*α*₂ agonist (*n*)	–	1	–

Data reported as mean ± SD. *n* = sample size.

**Figure 2:**
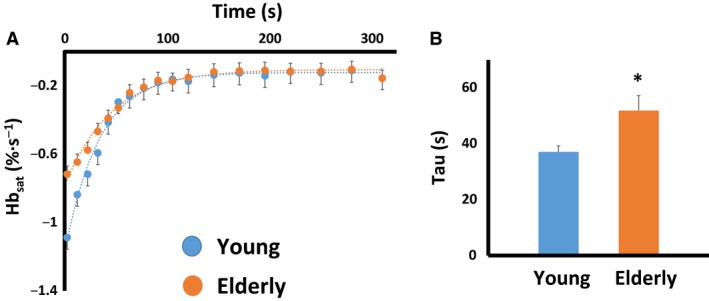
(A) Average postexercise muscle oxygen consumption recovery data for young and elderly participants. Note the slower rate of recovery in the elderly compared to the young, summarized in panel (B).

A typical NIRS recovery kinetics test is illustrated in Figure 1. Consistent with our hypothesis, the postexercise muscle oxygen consumption recovery kinetics — analogous to the recovery of phosphocreatine using ^31^P‐MRS (Ryan et al. 2013c) — were significantly prolonged in the elderly participants compared to younger individuals (51.8 ± 5.4 sec vs. 37.1 ± 2.1 sec, *P* = 0.04; Fig. 2). The average *r* ^2^ value of the fit monoexponential equation was 0.95, with values ranging from 0.91 to 0.99.

## Discussion

To the best of our knowledge, this is the first study to directly assess the effects of age on mitochondrial function using the rate constant for the recovery of mVO_2_ measured with NIRS. The major novel finding of this study was that even in nonlocomotor skeletal muscle, age significantly prolonged postexercise muscle oxygen consumption recovery kinetics. This data highlights an age‐related decline in muscle oxidative function and establishes the proof‐of‐concept for a new noninvasive, low‐cost methodology for future clinical application in gerontology.

Only one other study utilizing this specific NIRS approach has measured muscle oxidative function in elderly subjects (Southern et al. 2015); however, this study did not *directly* assess age‐related differences in muscle oxidative capacity (i.e., data were not compared to young control group). Instead, the elderly subjects studied in this prior investigation served as a healthy control group for a group of patients with heart failure with reduced ejection fraction. Moreover, the elderly participants included in this prior investigation were ~10 years younger than our elderly cohort. Despite modest differences in the type of forearm exercise performed and near‐infrared technology used, combining these two data sets portrays a progressive age‐related impairment in muscle oxidative function. Of note, our elderly participants appear closer to heart failure patients than young healthy controls or elderly participants ~10 years younger (Table 2).

**Table 2:** Comparison of the present muscle oxygen consumption recovery rate constant with previously published reports by the same technique in varying age groups

Reference/data set	Sample size (n)	Age of participants (years ± SD)	Tau (*τ*) (sec)
Present data (young)	17	25 ± 4	37
Southern et al. (2015) (elderly)	23	61 ± 5	38
Southern et al. (2015) (elderly + heart failure)	16	65 ± 7	46
Present data (elderly)	15	73 ± 4	52

That age was associated with a marked reduction in muscle oxidative function is consistent with previous studies using more established techniques (Coggan et al. 1993; McCully et al. 1993; Rooyackers et al. 1996; Conley et al. 2000; Short et al. 2005). For example, Conley et al. (2000) found aging to impair muscle oxidative function by ~50% compared to young subjects, which they attributed to both a decrease in mitochondrial volume and a lower oxidative function of the mitochondria. Unlike this prior investigation, however, the current data were obtained without the need for invasive muscle biopsies or expensive and time‐consuming MRS techniques. Like Conley et al. (2000), we interpret the slower recovery rate of mVO_2_ in our elderly participants to reflect an overall reduction in oxidative function.

The exact mechanism responsible for the observed age‐related decline in skeletal muscle oxidative function is beyond the scope of the present investigation, but is likely multifactorial. Aging has indeed been associated with a marked reduction in mitochondrial‐specific oxidative enzymes (Cooper et al. 1992; Boffoli et al. 1994; Rooyackers et al. 1996), mitochondrial DNA mutations (Cooper et al. 1992; Boffoli et al. 1994; Michikawa et al. 1999), oxidative damage by reactive oxygen species (Papa 1996), reduced synthesis of mitochondrial proteins (Rooyackers et al. 1996), and increased ATP and/or oxidative cost during exercise (Ferri et al. 2007; Layec et al. 2014). However, several studies have challenged the concept that aging itself affects muscle bioenergetics (Kutsuzawa et al. 2001; Carlson et al. 2008; Tevald et al. 2014), and instead suggest that muscle oxidative function is far more dependent on the muscle group studied (Kent‐Braun and Ng 2000; Lanza et al. 2005, 2007; Larsen et al. 2012) (locomotor vs. nonlocomotor) and/or the physical activity level of the individual (Brierley et al. 1996; Rasmussen et al. 2003; Rimbert et al. 2004; Larsen et al. 2012). That we specifically studied nonlocomotor muscle argues in favor of age‐specific changes in mitochondrial/oxidative function, but cannot completely rule this possibility out. Likewise, our results contradict that of Kutsuzawa et al. (2001), who failed to find age‐related differences in forearm phosphocreatine recovery kinetics using MRS. The elderly subjects studied herein however, were more than 10 years older (on average), with some history of cardiovascular risk factors. We therefore cannot rule out the possibility that age‐related declines in skeletal muscle oxidative function may not simply be a continuous variable, and is likely influenced by a variety of factors (absolute age, cardiovascular risk, and activity level). Regardless of mechanism, however, this study highlights the simplicity, robustness, and clinical utility of this novel, noninvasive spectroscopic approach, and demonstrates its usefulness in gerontology.

While NIRS does not directly measure skeletal muscle oxidative function, it has previously been validated against both in situ muscle biopsy measurements (Ryan et al. 2014a) and in vivo MRS (Ryan et al. 2013c) with excellent agreement. We did encounter several instances where this NIRS‐based approach was not successful. While half of these instances were attributed to nonbiological technical difficulties (i.e., patient compliance and instrument error), at least two instances appeared to be related to limb adiposity, which is known to affect NIRS (Ferrari et al. 2004). This will need to be accounted for if this technology is going to be incorporated into large clinical trials. Indeed, advancements in NIRS penetration depth, such as those previously described (Koga et al. 2015), may need to be considered. We also acknowledge that while our findings do not appear to be explained by the absence or presence of cardiovascular factors (i.e., hypertension or hypercholesterolemia) or specific medications used, we are in no way powered to fully test this relationship. Moreover, given the age and medical history of our elderly group, it is entirely possible that arterial stiffness may have influenced our results. Indeed, arterial stiffness can impact tissue hemoglobin oxygenation and therefore skeletal muscle oxidative recovery kinetics (Dipla et al. 2017). Because arterial stiffness was not measured in the present study, we cannot rule it out as a potential contributing factor.

In conclusion, we found that age was associated with a reduction in skeletal muscle oxidative function using a novel noninvasive and cost‐effective spectroscopic approach. Taken together, we believe this NIRS‐based approach holds great promise in gerontology (and clinical medicine in general) as a quantitative tool to assess skeletal muscle oxidative function at the bed side, in the clinic, and for the evaluation of therapeutic efficacy.

## Conflict of Interest

None declared.
